# Mapping N-linked glycosylation of carbohydrate-active enzymes in the secretome of *Aspergillus nidulans* grown on lignocellulose

**DOI:** 10.1186/s13068-016-0580-4

**Published:** 2016-08-08

**Authors:** Marcelo Ventura Rubio, Mariane Paludetti Zubieta, João Paulo Lourenço Franco Cairo, Felipe Calzado, Adriana Franco Paes Leme, Fabio Marcio Squina, Rolf Alexander Prade, André Ricardo de Lima Damásio

**Affiliations:** 1 Laboratório Nacional de Ciência e Tecnologia do Bioetanol (CTBE), Centro Nacional de Pesquisa em Energia e Materiais (CNPEM), Campinas, SP Brazil; 2 Department of Biochemistry and Tissue Biology, Institute of Biology, University of Campinas (UNICAMP), Rua Monteiro Lobato, 255, Cidade Universitária Zeferino Vaz, Campinas, SP 13083-862 Brazil; 3 Laboratório Nacional de Biociências (LNBio), Centro Nacional de Pesquisa em Energia e Materiais (CNPEM), Campinas, SP Brazil; 4 Department of Microbiology and Molecular Genetics, Oklahoma State University, Stillwater, OK USA

**Keywords:** Glycoproteomics, *Aspergillus nidulans*, Carbohydrate-active enzymes, CAZy, Glycoside hydrolases, N-glycosylation, Heterologous expression

## Abstract

**Background:**

The genus *Aspergillus* includes microorganisms that naturally degrade lignocellulosic biomass, secreting large amounts of carbohydrate-active enzymes (CAZymes) that characterize their saprophyte lifestyle. *Aspergillus* has the capacity to perform post-translational modifications (PTM), which provides an additional advantage for the use of these organisms as a host for the production of heterologous proteins. In this study, the N-linked glycosylation of CAZymes identified in the secretome of *Aspergillus nidulans* grown on lignocellulose was mapped.

**Results:**

*Aspergillus nidulans* was grown in glucose, xylan and pretreated sugarcane bagasse (SCB) for 96 h, after which glycoproteomics and glycomics were carried out on the extracellular proteins (secretome). A total of 265 proteins were identified, with 153, 210 and 182 proteins in the glucose, xylan and SCB substrates, respectively. CAZymes corresponded to more than 50 % of the total secretome in xylan and SCB. A total of 182 N-glycosylation sites were identified, of which 121 were detected in 67 CAZymes. A prevalence of the N-glyc sequon N-X-T (72.2 %) was observed in N-glyc sites compared with N-X-S (27.8 %). The amino acids flanking the validated N-glyc sites were mainly composed of hydrophobic and polar uncharged amino acids. Selected proteins were evaluated for conservation of the N-glyc sites in Aspergilli homologous proteins, but a pattern of conservation was not observed. A global analysis of N-glycans released from the proteins secreted by *A. nidulans* was also performed. While the proportion of N-glycans with Hex_5_ to Hex_9_ was similar in the xylan condition, a prevalence of Hex_5_ was observed in the SCB and glucose conditions.

**Conclusions:**

The most common and frequent N-glycosylated motifs, an overview of the N-glycosylation of the CAZymes and the number of mannoses found in N-glycans were analyzed. There are many bottlenecks in protein production by filamentous fungi, such as folding, transport by vesicles and secretion, but N-glycosylation in the correct context is a fundamental event for defining the high levels of secretion of target proteins. A comprehensive analysis of the protein glycosylation processes in *A. nidulans* will assist with a better understanding of glycoprotein structures, profiles, activities and functions. This knowledge can help in the optimization of heterologous expression and protein secretion in the fungal host.

**Electronic supplementary material:**

The online version of this article (doi:10.1186/s13068-016-0580-4) contains supplementary material, which is available to authorized users.

## Background

The dependence on energy sources derived from fossil fuels and the environmental impact caused by their use have generated special interest from researchers and governments regarding the use of renewable energy sources. The use of renewable sources for fuel production has become an important alternative because they generate fewer pollutants and allow the sustainable development of the economy and human society. Alternatively, the use of lignocellulosic biomass, mainly composed of cellulose, hemicellulose and lignin, is a consensus worldwide because it is the most abundant renewable energy source on Earth [1]. However, the use of this biomass in the biorefinery concept requires its depolymerization to mono- and oligosaccharides, which are the building blocks used to produce biofuels and biochemicals.

Plant biomass is a complex structure rich in glycoconjugates and poly- and oligosaccharides, and a wide variety of enzymes are necessary for the complete degradation of this biomass [2, 3]. Carbohydrate-active enzymes (CAZymes) participate in the breakdown, biosynthesis and modification of the glycoconjugates and oligo- and polysaccharides that constitute the plant cell wall. In general, CAZymes are structurally constituted by a catalytic domain, and some CAZy families have an additional carbohydrate-binding module (CBM). Based on structural and homology features, the CAZy database currently covers five enzyme classes, including glycoside hydrolases (GHs), glycosyltransferases (GTs), polysaccharide lyases (PLs), carbohydrate esterases (CEs) and auxiliary activities (AAs) [4].

The genus *Aspergillus* includes microorganisms that naturally degrade lignocellulosic biomass and secrete large amounts of CAZymes, which characterize their saprophyte lifestyle [5]. This complex biomass is partially degraded, releasing simple carbohydrates that are readily taken up by the fungal cells to provide energy for their growth and reproduction. Due to this capacity for secretion of a large amount and variety of enzymes, along with the abilities to tolerate extreme cultivation conditions in liquid- and solid-state fermentation, the *Aspergillus* fungus has been a successful model for enzyme production on an industrial scale [6].

*Aspergillus* has the capacity to perform post-translational modifications (PTM) such as proteolytic cleavage, disulfide bond formation and glycosylation of proteins, providing an additional advantage for the use of these organisms as a host for the production of heterologous proteins [7]. Asparagine-linked protein N-glycosylation is a prevalent PTM in eukaryotic systems, and has also been described in prokaryotic systems [8]. The N-glycosylation consists of the co- or post-translational attachment of an oligosaccharide to proteins by covalent bonds in the endoplasmic reticulum (ER) lumen [9, 10]. N-glycosylation of proteins is essential for a range of cellular processes such as immune responses, cellular communication, intracellular trafficking, stability, secretion, folding and protein activity [10–13]. In eukaryotes, N-linked glycosylation occurs at the Asn-X(aa)-Ser/Thr sequon and is a co-translational process catalyzed by oligosaccharyltransferases (OST) in the lumen of the ER [8].

Glycoscience, which involves N-glycosylation studies that have been performed primarily for an understanding of the role of carbohydrates on biophysical modifications in cell communication, is aimed at developing new approaches for the treatment of human diseases [14–16]. However, some recent studies have shown the effect of glycosylation on folding, secretion and enzymatic properties [17]. Knowledge of N-glycosylation of CAZymes is scarce and mainly reported for cellobiohydrolases [18–20]. The correct glycosylation of proteins becomes an essential feature in systems for the heterologous expression of target genes using filamentous fungi as a host because the accumulation of unfolded or misfolded proteins is a bottleneck in the secretion pathway and also in the protein production yield [11, 21].

Accumulation of misfolded proteins overloads the ER processing capacity, triggering a response called the unfolded protein response (UPR). The UPR pathway activates a large set of genes responsible for correct protein folding, degradation of misfolded proteins and others to recover proteostasis [22, 23] Thus, larger amounts of proteins acquire the correct folding, can leave the ER bound for the extracellular environment and are not targeted for degradation. The decrease in glycosylation levels by reducing the expression of oligosaccharyltransferase genes leads to cell stress conditions. ER stress induced by the low levels of glycosylation of some proteins leads to the overexpression of several UPR genes, including genes related to cell wall biogenesis, protein folding and degradation of unfolded proteins [24].

There are a few studies mapping the global N-glycosylation of CAZymes in filamentous fungi [25, 26]. In this study, the N-linked glycosylation of CAZymes identified in the secretome of *Aspergillus nidulans* grown on lignocellulose was mapped. Therefore, *A. nidulans* was grown in glucose, xylan and pretreated sugarcane bagasse (SCB), followed by glycoproteomics and glycomics on the extracellular proteins (secretome). The most common and frequent N-glycosylated motifs, an overview of CAZymes’ N-glycosylation and the number of mannose residues found in N-glycans were analyzed. A comprehensive analysis of protein glycosylation processes in *A. nidulans* will assist with a better understanding of glycoprotein structures, profiles, activities and functions. This knowledge can help in the optimization of heterologous expression and protein secretion in the fungal host.

## Results

### Prediction of N-glycosylated CAZymes in the *Aspergillus nidulans* genome

To identify all the putative *A. nidulans* glycoproteins involved in lignocellulose degradation, a comprehensive analysis of the *A. nidulans* ORFs (10,678 entries) downloaded from the *Aspergillus* Genome Database (AspGD) was performed [27]. First, 428 CAZymes (4 % of the *A. nidulans* ORFs) were annotated by dbCAN (automated CAZymes annotation) [28]. Second, 359 out of 428 CAZymes were predicted to contain at least one N-glycosylation site (N-glyc site) by the NetNGlyc 1.0 Server. Finally, the predicted N-glycosylated CAZymes were analyzed for the presence of signal peptide cleavage sites using the SignalP 4.1 Server (Additional file 1: Figure S1).

The majority (73 %) of the 190 N-glycosylated CAZymes identified with signal peptide were classified as glycoside hydrolases (GHs), and 7 % had a C-terminal-associated carbohydrate-binding module (CBM). The other CAZymes were predicted as auxiliary activities (AAs; 9 %), carbohydrate esterases (CEs; 7 %), polysaccharide lyases (PLs; 7 %) and glycosyltransferases (GTs; 4 %). The number of predicted N-glyc sites in the 190 secreted CAZymes varied from 1 to 21 sites and approximately 40 % of proteins had one or two N-glyc sites (Additional file 1: Figure S2).

### Proteomics overview of *A. nidulans* grown on glucose, xylan and alkali pretreated sugarcane bagasse (SCB)

**Fig. 1:**
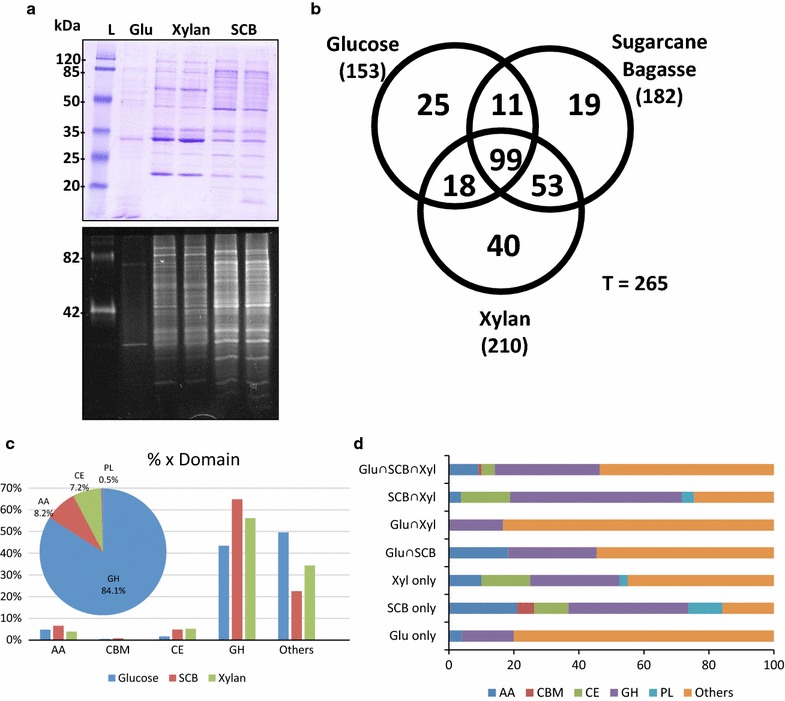
Overview of secretomes from *A. nidulans* grown on glucose, xylan and sugarcane bagasse. **a** *Aspergillus nidulans* secretomes were stained with coomassie brilliant blue and Pro-Q Emerald for detection of glycoproteins in polyacrylamide gels. *L* ladder, *Glu* glucose, *SCB* sugarcane bagasse. The secretomes produced on xylan and SCB are represented in duplicate; however, the experiments were performed in triplicate. **b** Mascot searches were carried out using the *Aspergillus* Genome Database (AspGD). The data were analyzed by the Scaffold software and the Venn diagram represents the number of proteins identified in each secretome. **c** Abundance of CAZymes classes identified in each condition. **d** CAZymes diversity shared among the growth conditions. The intersection symbol “∩” means that proteins are common in two or more conditions. *GH* glycoside hydrolases, *PL* polysaccharide lyases, *CE* carbohydrate esterases, *AA* auxiliary activities, *CBM* carbohydrate-binding module

*Aspergillus nidulans* was grown in three different substrates for 96 h (glucose, xylan and SCB in biological triplicates), and the secretomes were evaluated by SDS-PAGE stained with Coomassie blue for the total protein profile and with Pro-Q Emerald for glycoprotein detection (Fig. 1a).

To identify the glycoproteins occurring in each cultivation condition, the secretomes were first enriched by ConA and then analyzed by LC–MS/MS. A total of 265 proteins was identified using one unique peptide and 0.1 % of FDR (False Discovery Rate). For the glucose, xylan and SCB conditions, 153, 210 and 182 proteins were identified, respectively, with 99 proteins common to all conditions (Fig. 1b). CAZymes corresponded to more than 50 % of the total secretome in the xylan and SCB conditions, and 59, 111 and 107 CAZymes were identified in the glucose, xylan and SCB conditions, respectively, and (Additional file 2: Table S1). Glycoside hydrolase was the most abundant class followed by AAs and CEs (Fig. 1c). In the glucose condition, 61.5 % of the proteins were assigned as non-CAZymes. In addition, a high variation in the proportion of CAZyme classes and families throughout the three substrates was observed, with *A. nidulans* secreting the highest diversity of CAZymes in the SCB condition. A further examination of the proteins that were exclusively identified in the SCB condition showed a high abundance of GHs linked to CBMs. These results are directly associated with the greater complexity of sugarcane bagasse and thus the requirement for a higher range of enzymes to degrade it (Fig. 1d).

**Fig. 2:**
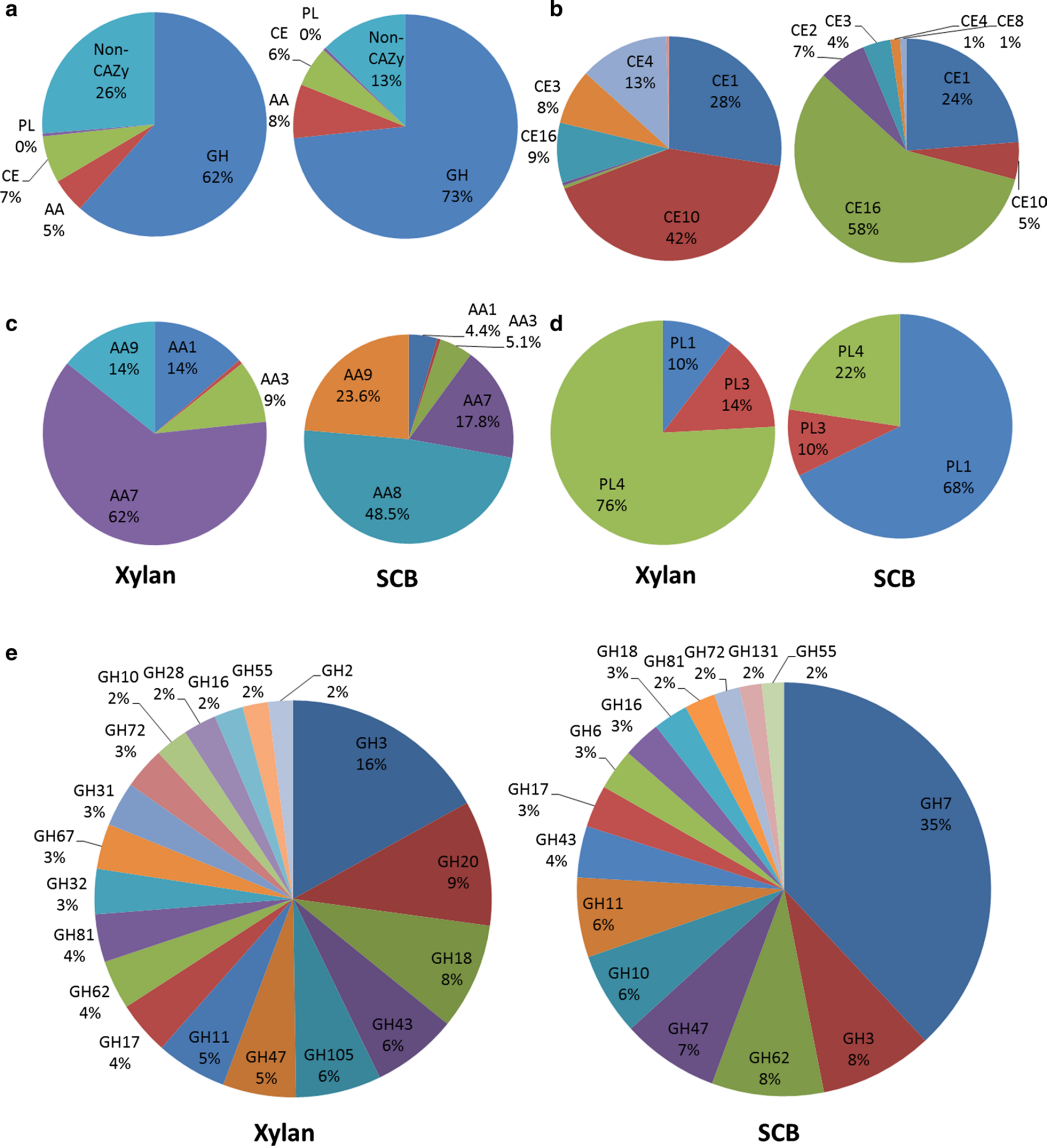
CAZymes annotation in the secretomes of *A. nidulans* grown on xylan and sugarcane bagasse. CAZymes were annotated by an HMM-based database (dbCAN). **a** Proteins were grouped in CAZy and Non-CAZy, and the CAZymes were grouped according to enzyme classes in carbohydrate esterases-CEs (**b**), auxiliary activities-AAs (**c**), polysaccharide lyases-PLs (**d**) and glycoside hydrolases-GHs (**e**). The total number of proteins in each class of enzyme was set as 100 % and families representing less than 2 % of the total proteins were not shown. *SCB* alkali–—pretreated sugarcane bagasse

In addition to the variations in the proportions of the CAZyme classes among the substrates (Fig. 2a), different compositions were also observed at the family level (Fig. 2b–e). Regarding the CEs, family CE16, known as carbohydrate acetylesterases active on various carbohydrate acetyl esters, was the most abundant in the SCB condition, representing 58 %, followed by CE1 (feruloyl esterases) and CE2 (acetyl xylan esterases). In the xylan condition, family CE10 was the most representative, although the members of this family are esterases that act on non-carbohydrate substrates [29]. Thus, the most abundant carbohydrate esterase family was CE1 (28 %), a classical family of feruloyl esterases, followed by CE4 (acetyl xylan esterases) and CE16 (acetyl esterases) (Fig. 2b).

The AA8 (flavocytochrome–cellobiose dehydrogenases) and AA9 (former copper-dependent lytic polysaccharide monooxygenases—LPMOs) families were the most abundant oxidative enzymes in the SCB condition, at 48.5 and 23.7 % of the total AAs, respectively. Moreover, AA8 was exclusively reported in the SCB condition. Both enzyme families are highly correlated with the oxidative degradation of cellulose in fungi. However, in the xylan condition, the most representative families were AA7 (oligosaccharide oxidases), AA1 (laccases) and AA9 (LPMOs) (c). Among the PLs, family PL1 (pectin lyase) was the most abundant in the SCB condition (68 %), followed by PL4 (rhamnogalacturonan lyase), whereas PL4 was the most representative family in the xylan condition at 76 %, followed by PL3 (pectate lyase) (Fig. 2d). The analysis of spectrum counts showed that GH7, a family of cellobiohydrolases/exoglucanases, was the most abundant GH family in the SCB condition, which represented 35 % of the GHs, followed by GH62 (α-l-arabinofuranosidase) and GH3 (β-gluco/xylosidase). Families such as GH5 (endo-glucanases) and GH6 (exo-glucanases) were exclusively identified in the SCB condition (Fig. 2e). GH3 was the most abundant family in the xylan condition, accounting for 16 % of the total spectrum counts of GHs, followed by GH20 (β-hexosaminidase), GH18 (chitinase) and GH43 (xylanase and α-l-arabinofuranosidase) (Fig. 2e).

**Fig. 3:**
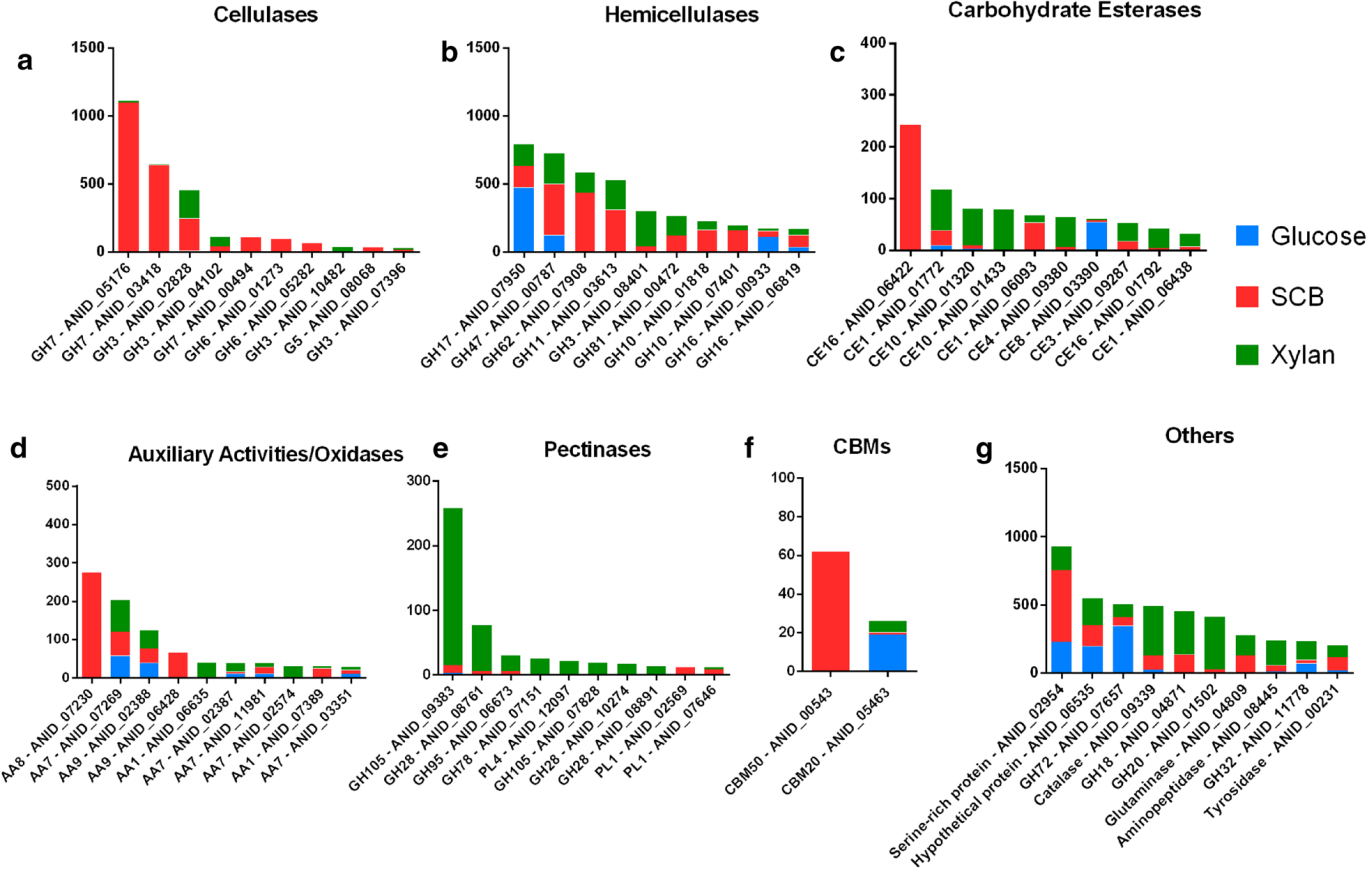
Top ten proteins secreted by *A. nidulans*. The total spectrum counts of a specific protein were summed and grouped in according to their functions. **a** cellulases, **b** hemicellulases, **c** carbohydrate esterases, **d** auxiliary activities/oxidases, **e** pectinases, **f** CBMs and **g** other functions

At the individual protein level, a GH7 cellobiohydrolase (ANID_05176) was the most abundant protein identified, accounting for 1098 peptides in the SCB condition (Fig. 3a). The GH3 (ANID_02828) was the most secreted β-glucosidase and showed the same spectrum counts in the xylan and SCB conditions (Fig. 3a).

In addition, GH47 α-mannosidase (ANID_00787) and GH62 α-l-arabinofuranosidase were also represented, both with more spectrum counts in the SCB condition than in the xylan or glucose conditions. One GH11 (ANID_03613) and two GH10 xylanases (ANID_01818; ANID_07401) were also more abundant in the SCB condition than in the xylan condition.

CE16 acetyl esterase (ANID_06422) was the most abundant CE found in the SCB condition, whereas *A. nidulans* secreted primarily two CE10 (ANID_01320; ANID_01433) in the xylan condition. Considering all conditions, an AA8 cellobiose dehydrogenase (ANID_07230) was the most abundant AA but was only detected in the SCB condition. The AA7 gluco-oligosaccharide oxidase (ANID_07269) and AA9 LPMO (ANID_02388) were the most “regular” enzymes, with equal secretion levels in all the conditions. However, the AA9 LPMO (ANID_06428) had peptides only reported in the SCB condition.

All the pectinases and polysaccharide lyases were more secreted in the xylan condition by far than in the SCB or glucose conditions. The GH105 rhamnogalacturonyl hydrolase (ANID_09383) was the most abundant pectinase, followed by the GH28 exo-polygalacturonase (ANID_08761), both showing high levels of secretion in the xylan condition. The PL4 rhamnogalacturonan lyase PL4 (ANID_12097) was the most abundant enzyme among the PLs.

Among the enzymes classified as “others”, a serine protease (ANID_02954) was the most abundant enzyme in SCB, followed by a hypothetical protein (ANID_06535) and a chitinase (ANID_04871). In the xylan condition, the most representative enzymes were *N*-acetylglucosaminidase (ANID_01502) followed by catalase (ANID_09339) and chitinase (ANID_04871).

The enzymatic activities in the *A. nidulans* secretomes were also analyzed (Additional file 1: Figure S3). The highest activity was detected on β-glucan and xylan using the SCB secretome. Using the xylan secretome, the highest activity was reported on xylan from beechwood, followed by β-glucan and mannan. Non-significant activities were found using the glucose-condition secretome. All these enzymatic activities were in accord with the proteome profile found for each growth condition as described above.

### N-glycosylated sites detected on CAZymes

As previously mentioned, 265 proteins were detected by LC–MS/MS after enrichment by ConA, and at least one N-glyc site was confirmed in 103 proteins. Considering all the N-glyc sites predicted by the NetNGlyc server, we defined three groups of sites in this work: (1) validated sites: N-glyc sites confirmed by our LC–MS/MS data set using the Mascot v.2.3.01 engine with GlcNAc tagged on an asparagine residue (N + 203) as a variable modification; (2) non-validated sites: N-glyc sites not confirmed by the LC–MS/MS. Then, these sites are non-glycosylated based on our data. (3) non-covered sites: peptides with this specific N-glyc site were not detected by the LC–MS/MS data.

A total of 182 N-glyc sites were validated, of which 121 were detected in 67 CAZymes (Additional file 3: Table S2). Table 1 shows the validated N-glyc sites of selected CAZymes. The AA8 cellobiose dehydrogenase (ANID_07230) was predicted to contain six N-glyc sites but we validated five of them (N132, N299, N308, N620 and N709). Only one N-glyc site (N679) was not covered in our data set. The protein ANID_02828 was the highest GH3 β-glucosidase secreted by *A. nidulans* in the xylan and SCB conditions. Two out of three predicted N-glyc sites in ANID_02828 were validated, N225 and N365, but the peptide glycosylated at N340 was not covered.

**Table 1:** Total spectrum count and N-glycosylation sites of selected CAZymes

Accession number	Identified proteins	N-glycosylated sites	CAZy	Total spectrum count		
				Glucose	SCB	Xylan
Auxiliary activities (AAs)						
ANID_07812	Conserved hypothetical protein	444, 501	AA3	0	23	1
ANID_02574	Conserved hypothetical protein	212, 330	AA7	0	1	29
ANID_07269	Conserved hypothetical protein	133, 460	AA7	58	63	82
ANID_02387	FAD binding domain-containing protein	260	AA7	11	5	23
ANID_07230	Cellobiose dehydrogenase	132, 299, 308, 620, 709	AA8	0	275	0
ANID_02388	Conserved hypothetical protein	93	AA9	39	38	47
ANID_06428	Fungal cellulose binding domain-containing protein	69	AA9	0	67	0
Carbohydrate esterases (CEs)						
ANID_06438	Dipeptidyl-peptidase IV	496, 671	CE1	0	7	25
ANID_06093	Acetylxylan esterase	263	CE1	0	53	15
ANID_01433	Triacylglycerol lipase	374, 381	CE10	0	2	77
ANID_01320	Conserved hypothetical protein	63	CE10	3	7	70
ANID_09130	Cholinesterase	79	CE10	2	8	14
Glycoside hydrolases (GHs)						
ANID_07401	Endo-1,4-β-xylanase	123	GH10	0	160	37
ANID_11143	Glucoamylase	428	GH15	1	10	9
ANID_08761	Exo-polygalacturonase	113, 199, 292, 297	GH28	0	6	71
ANID_04102	β-Glucosidase	62, 491, 642, 713	GH3	1	39	73
ANID_08401	β-Xylosidase	63, 340, 408, 419	GH3	0	40	261
ANID_02828	β-Glucosidase	225, 365	GH3	8	238	208
ANID_07275	Xylosidase/glycosyl hydrolase	40, 382	GH43	0	55	15
ANID_08007	Endo-α-1,5-arabinanase	126	GH43	12	38	118
ANID_08477	Arabinofuranosidase	438	GH43	0	20	27
ANID_05176	1,4-β-d-Glucan-cellobiohydrolyase	284	GH7	0	1098	15
ANID_00472	Endo-1,3-β-glucanase Engl1	219, 240	GH81	0	120	147
Polysaccharide lyases (PLs)						
ANID_12097	Rhamnogalacturonan lyase	231	PL4	0	0	22

We performed an additional validation of the N-glyc sites using the Scaffold PTM software with default statistical parameters pre-established by the program, based on the presence and intensity of site-specific ions compared randomly [30]. From the 182 sites previously validated, 151 sites were re-validated by the additional statistical filters, which increased the sensitivity to peptide spectra matches [30]. We further analyzed the 151 re-validated N-glyc sites to determine if there was a specific amino acid motif surrounding the N-glyc sites. The amino acid sequence of all validated N-glycopeptides was aligned, and six amino acid residues before and after the sequons (N-X-S/T) were analyzed. The prevalence of the sequence N-X-T (72.2 %) over N-X-S (27.8 %) was observed. Furthermore, the sequon N-X-T showed additional motif variations (Table 2; Additional file 4: Table S3). In addition to the 182 N-glyc sites validated, 23 predicted N-glyc sites were not confirmed. The sequon N-X-S (60.9 %) was predominant for those non-validated sites as opposed to the validated sites.

**Table 2:** Motifs report for the flanking sequences dataset of N-glycosylated sites

Motif	Dataset matches^a^	Dataset percentage^b^	Background percentage^c^
……n.S….	42	27.8	6.60
……n.T….	109	72.2	7.30
……nGT…	19	12.6	1.50
……nTT….	12	7.90	0.92
……nST….	14	9.30	0.69
……n.T.T..	15	9.90	0.96
…P..n.T….	15	9.90	0.80
T…..n.T….	14	9.30	0.92

*S* serine, *T* threonine, *P* proline

^a^The data set consists of the flanking sequences for N-glycosylated sites

^b^Dataset percentage was calculated based on the probability that a modification occurs in a given motif throughout all identified peptides

^c^Background percentage was calculated using all proteins loaded into the program as background and measuring the probability that a specific amino acid appears with a motif. The dots correspond to the amino acids flanking glycosylated asparagines (n)

**Fig. 4:**
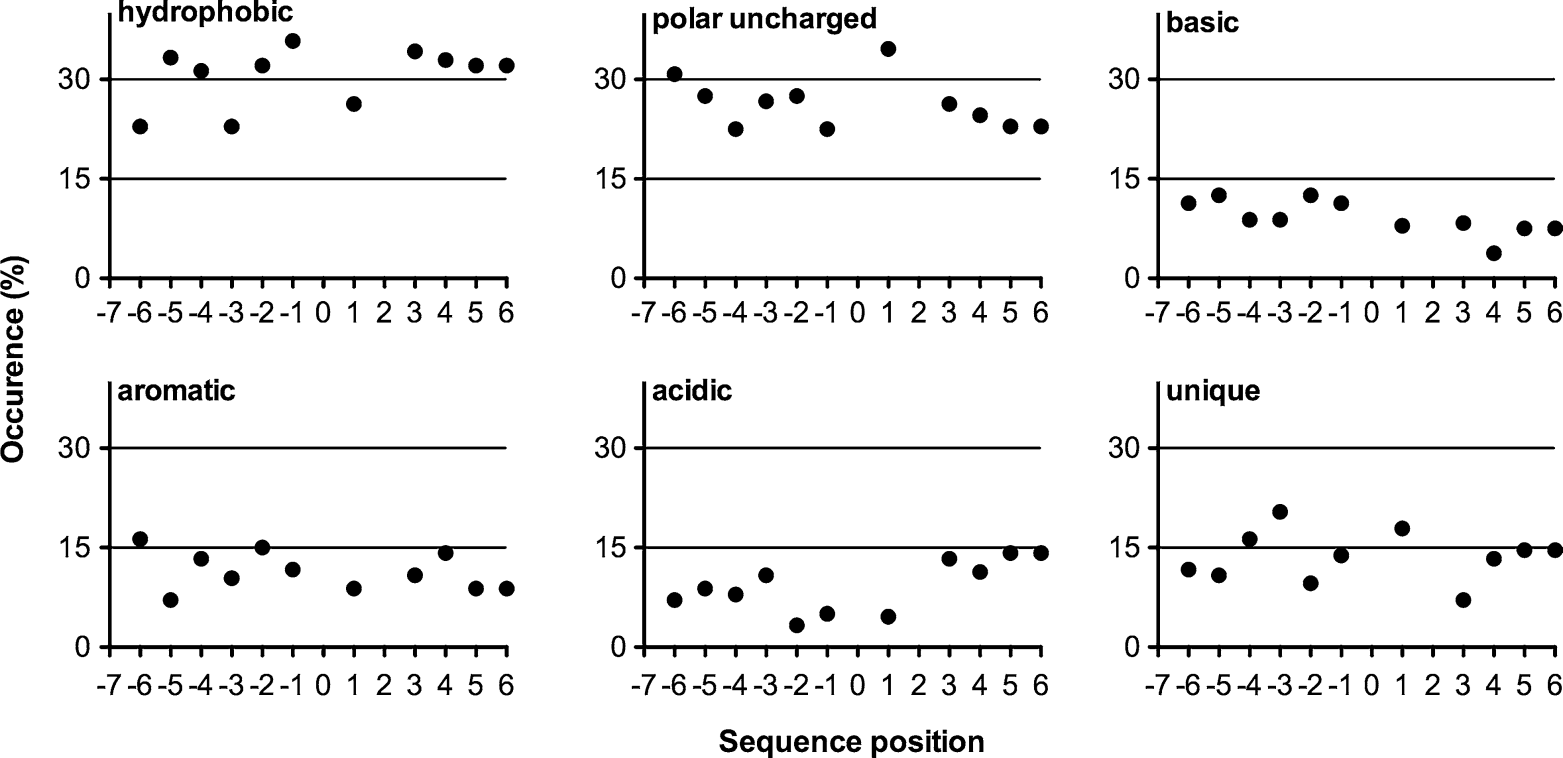
Amino acids flanking validated N-glycosylation sites. The relative occurrence of amino acids is plotted versus sequence position −6 to +6 around an occupied N-glyc site. Residues specified by the glycosylation sequon (0 = Asn; +2=Ser or Thr) are not plotted. Hydrophobic (Ala, Val, Leu, Ile, Met); Aromatic (Phe, Tyr, Trp); Polar uncharged (Ser, Thr, Asn, Cys, Gln); Acidic (Asp, Glu); Basic (Lys, Arg, His); Unique (Gly, Pro)

The amino acids flanking the validated N-glyc sites (from −6 to +6) were classified according to the chemical properties of the side chains (Fig. 4). These flanking regions were mainly composed by hydrophobic and polar uncharged amino acids. However, this profile was different for the non-validated N-glyc sites (Additional file 1: Figure S4).

### Conservation of N-glycosylated sites in *Aspergilli*

**Fig. 5:**
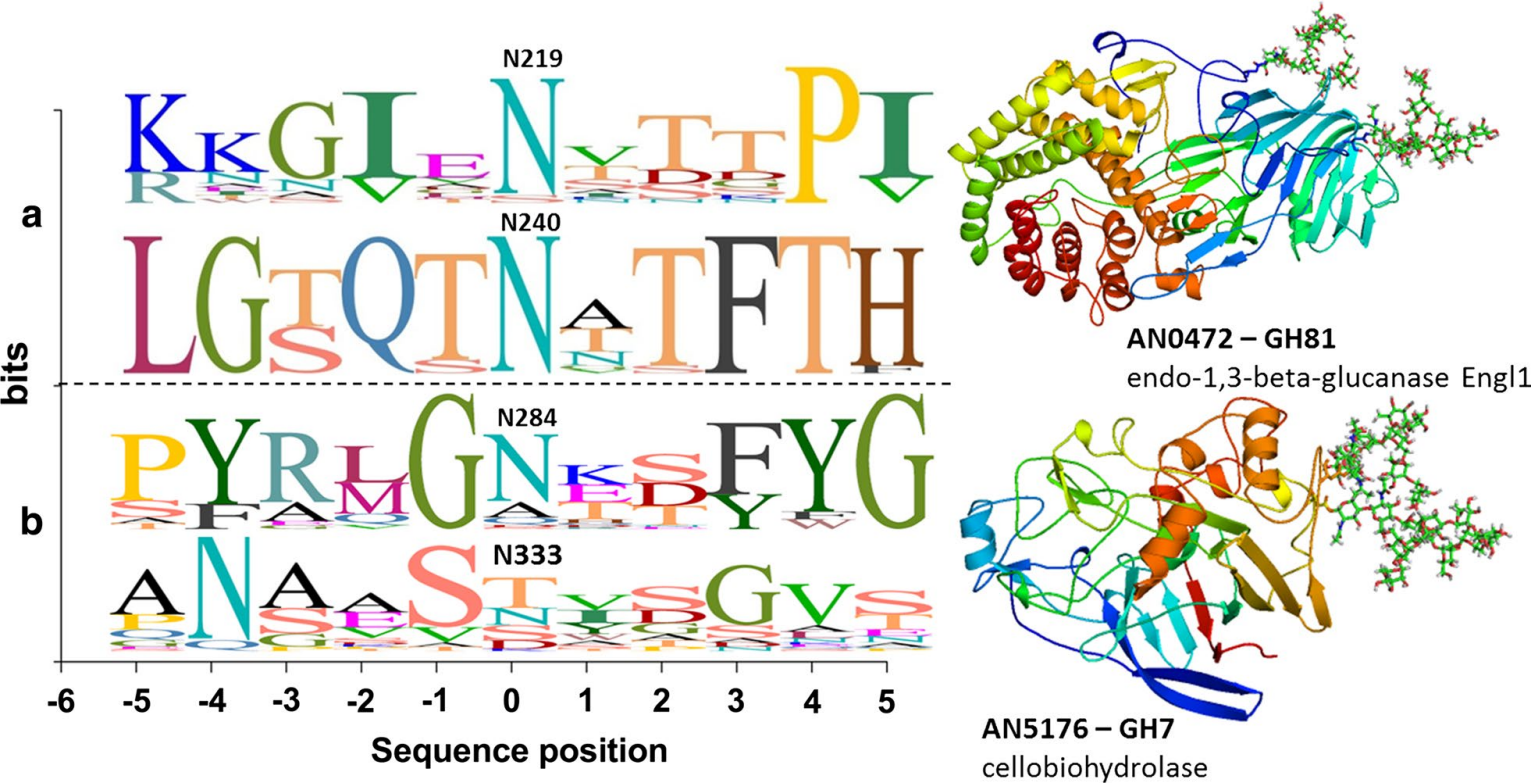
Conservation of glycosylation sites in selected proteins identified on sugarcane bagasse-secretome. N-glyc sites of selected proteins were analyzed by conservation in homologous proteins on AspGD. **a** AN0472 is a GH81 endo-1,3-β-glucanase Engl1 secreted on xylan and SCB. The protein model was based on PDB 4K3A. **b** AN5176 is a GH7 cellobiohydrolase highly secreted in SCB. The protein model was based on PDB 1Q9H

To investigate if the majority of N-glyc sites were conserved in homologous proteins, we aligned selected proteins from *A. nidulans* with 19 *Aspergilli* genomes from the AspGD (Table 3). Proteins were considered homologous when the *E* value (Blastp) was equal to or less than 1.00E−70. Two CAZyme sequences are shown in Fig. 5. The ANID_00472 is a GH81 endo-1,3-β-glucanase Engl1 that was secreted by *A. nidulans* in the xylan and SCB conditions. Four N-glyc sites were predicted for this protein (N219, N240, N257, N499), two of which were validated by the glycoproteomics. We found and aligned 20 sequences homologous to this GH81, generating a sequence logo. The N-glyc sites N219 and N240 were highly conserved throughout all the homologous sequences, 90 and 95 %, respectively. Similarly, ANID_05176 is a GH7 cellobiohydrolase with two predicted N-glyc sites (N284 and N333), but the N333 was non-glycosylated according to our data. We found and aligned 50 homologous sequences, and while the N284 site was conserved in 62 % of the homologous sequences, the N333 was present in only one homologous sequence. The conservation of N-glyc sites ranges from 8 to 100 % (Table 3) and, therefore, there was no pattern of conservation of N-glyc sites in Aspergilli homologous sequences.

**Table 3:** Conservation of N-glycosylated sites of selected *A. nidulans* CAZymes in homologous proteins

Identified proteins	Accession number	Domain	N-glyc sites^a^	Conservation (%)^b^	Homologous in *Aspergilli* ^*c*^	Sequon^d^
Endo-1,3-β-glucanase Engl1	ANID_00472	GH81	219	90.0	20	NSS
			240	95.0	20	NAT
α-Glucosidase AgdA	ANID_02017	GH31	432	91.3	22	NAS
β-Xylosidase	ANID_02359	GH3	231	43.3	30	NHS
			673	55.9	34	NFT
			695	52.9	34	NTT
β-Glucosidase	ANID_02828	GH3	225	100	50	NGT
			365	34.0	50	NGS
β-Glucosidase	ANID_04102	GH3	62	98.0	50	NLT
			491	8.0	50	NKT
			642	92.0	50	NQT
			713	40.0	50	NST
1,4-β-d-Glucan-cellobiohydrolyase	ANID_05176	GH7	284	62.0	50	NTS
Cellobiose dehydrogenase	ANID_07230	AA8	132	37.5	24	NAT
			299	47.4	38	NGT
			308	90.0	40	NGT
			620	7.9	38	NVT
			709	23.7	38	NVS
β-Glucosidase	ANID_07396	GH3	259	100	50	NNS
			438	72.0	50	NGT
			586	56.0	50	NSS
Endo-1,4-β-xylanase	ANID_07401	GH10	123	100	11	NTT
β-Xylosidase	ANID_08401	GH3	63	70.0	30	NNT
			340	30.0	30	NET
			408	46.7	30	NGT
			419	93.3	30	NFT
Arabinofuranosidase	ANID_08477	GH43	438	13.6	23	NGS
Exo-polygalacturonase	ANID_08761	GH28	113	62.1	29	NDT
			199	72.4	29	NSS
			292	100	30	NIS
			297	63.3	30	NAS
Exopolygalacturonase	ANID_08891	GH28	65	21.9	32	NDT
			230	94.1	17	NAS
α-Glucuronidase	ANID_09286	GH67	48	85.0	20	NAT
			315	95.0	20	NRT
			689	90.0	20	NKS
			769	15.0	20	NST
Exo-rhamnogalacturonase B	ANID_10274	GH28	34	53.3	15	NET
			340	100	16	NCT
β-Glucosidase	ANID_10482	GH3	73	100	50	NLT
			726	60.0	50	NSS

^a^N-glycosylated sites confirmed in the *A. nidulans* proteins by LC–MS/MS

^b^Alignment gaps on the sequences shift the N-glycosylation sequons in homologous sequences. Despite the sequons are in different positions on primary sequence, the N-glycan is attached to similar positions at proteins 3D-level (see Fig. 7)

^c^Proteins were considered homologous when the *E* value (Blastp) was equal to or less than 1.00E−70

^d^Sequon detected in *A. nidulans* glycoproteins by LC–MS/MS

### Profile of N-glycans attached to proteins secreted by *A. nidulans*

**Fig. 6:**
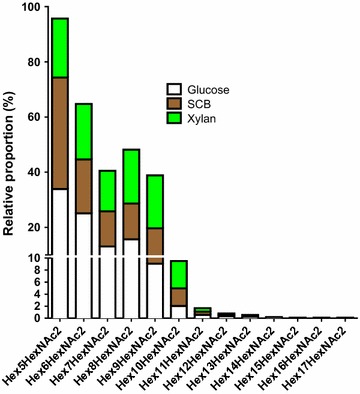
N-glycans profiling of glycoproteins secreted by *A. nidulans* cultured in glucose, sugarcane bagasse and xylan. About 500 µg of total secretome was incubated with PNGaseF at 37 °C overnight to release N-glycans. N-glycans were permethylated and profiled by MALDI/TOF–MS

A global analysis of N-glycans released from proteins secreted by *A. nidulans* was also performed (Fig. 6). Mannose and galactofuranose are structural isomers, have exactly the same mass and are non-distinguishable in MALDI/TOF–MS oligosaccharide profiling. Thus, the peaks were labeled as “Hex_5_HexNAc_2_” instead of “Man_5_GlcNAc_2_”. The relative proportion of each N-glycan component was slightly different in the samples (Additional file 5: Table S4) [31–33]. While the proportion of the N-glycans with Hex_5_ to Hex_9_ was similar in the xylan condition, a prevalence of Hex_5_ was observed in the SCB and glucose conditions. Moreover, traces of N-glycans with Hex_14_ to Hex_17_ were only detected in the glucose condition.

## Discussion

### The enzymatic repertoire secreted by *A. nidulans* matches well to the composition of the substrate

The N-glycoproteomic analysis of *A. nidulans* secretomes revealed that the substrate composition and architecture directly influenced the abundance and repertoire of CAZymes. Our data suggested that sugar monomers and oligomers from xylan and SCB induced the secretion of a complete repertoire of enzymes by *A. nidulans*. Delmas et al. [34] reported the transcriptional response of *A. niger* to complex substrates. In general, when *A. niger* is under starvation (no carbon source) the transcription factor CreA, which act as glycoside hydrolases transcription repressor, is derepressed allowing a basal expression of a set of GHs. After the initial uptake of mono and oligosaccharides by the fungus, the transcription factor XlnR is activated inducing the transcription of several CAZymes [34]. Souza and Gouveia [35] also reported a complex transcriptional response of *A. niger* grown on sugarcane bagasse. Moreover, the transcriptional response of ascomycetes to complex substrates involves others activators, such as AmyR, InuR, AraR, GalR, GalX and RhaR [36].

In the xylan condition, the majority of proteins identified in the secretome were correlated with xylan and xylooligosaccharides degradation, as previously reported for *A. fumigatus* [37]. GH3 was the main family identified in this condition, which is consistent with the secretome of *Penicillium purpurogenum* grown on acetylated xylan [38]. The identified GH3 enzymes were annotated as β-xylosidases and β-1,3/1,4-glucosidases (Additional file 2: Table S1), suggesting xylose production and uptake by *A. nidulans*. In a secretome of *A. fumigatus* grown on xylan, the major families identified were GH10 and GH11, followed by GH3 [37].

Moreover, there was a higher abundance of peptides from families GH20 and GH18 in the xylan condition, and both families are related to cell wall degradation/remodeling, as well as to protein synthesis/degradation enzymes such as glutaminases, tyrosinases and proteases [39, 40]. In addition, a high abundance of catalase was found in the xylan condition, an enzyme related to fungal growth and hyphae development [41]. A set of proteins related to fungi growth and development was also found in the SCB condition, which was consistent with the secretome of *A. nidulans* grown on sorghum stover [42]. Adav et al. [37] also reported a high abundance of esterases in the secretome of *A. fumigatus* grown on xylan.

GH7 has been described as the major enzyme secreted by fungi to degrade cellulose and complex lignocellulose [2, 3]. GH7 proteins were the most abundant enzymes in the secretome of *A. nidulans* grown on sorghum stover [42] and in the secretome of *Trichoderma reesei* grown on sugarcane culms and bagasse, which, along with GH6, accounted for 80 % of the peptide counts [43]. Ribeiro et al. [44] reported that the GH7 family, along with GH5 and GH6, represented the most important set of enzymes secreted by *Penicillium echinulatum* grown on integral and pretreated sugarcane bagasses, as well as on pure cellulose.

The highest abundance of hemicellulase peptides was identified in the SCB condition compared with the xylan condition (Additional file 2: Table S1), such as GH3, GH62, GH10 and GH11. This result was further validated by enzymatic activity assays with *A. nidulans* secretomes, which reported higher hemicellulase activity in the SCB-derived secretome than in the xylan secretome (Additional file 1: Figure S3). The known composition of SCB is 60 % cellulose, 23 % hemicelluloses, 8 % lignin and 10 % ashes, which suggest that *A. nidulans* requires the secretion of different hemicellulases aimed at detaching the xylan from the cellulose. Thus, the recalcitrant cellulose from SCB could be accessed and degraded by a set of cellulases and oxidative enzymes.

LPMOs from families AA9 and AA10 have been reported as the major enzymes for boosting lignocellulose breakdown in commercial cellulase cocktails [45, 46]; however, little is known regarding their biological role in fungal and bacterial physiology [47, 48]. These enzymes require an electron donor to oxidize lignocelluloses, which can be donated through a non-enzymatic donor, such as lignin or a reducing agent, or using enzymes such as cellobiose dehydrogenases (CDH) and oligosaccharide oxidases [47, 48]. Our results showed that the AA8 family (CDH) members were secreted only in the SCB condition, whereas AA3 (GOOX glucooligosaccharide oxidases) was reported in all conditions. These results suggest that these enzymes were differentially regulated in response to substrate in *A. nidulans* [49]. AA7 (GOOX) enzymes were the most abundant AA in the xylan condition. This family has been reported to be GOOX capable of oxidizing the reducing end of glycosyl residues of oligosaccharides [50]. Members of family AA7, along with AA3, have been described as enzymes that generate hydrogen peroxide as a co-product of the reactions they are involved in, which can act as a co-factor for AA1 laccases, such as ANID_06635, another enzyme described in high abundance in the xylan condition [51]. However, catalase B (ANID_09339) was also described in high abundance in the xylan and SCB conditions. This type of enzyme is well known to decompose hydrogen peroxide in biological systems. Thus, we suggest that there was a fine control of this reactive oxygen species in the secretome, as H_2_O_2_ could be used as a co-factor for laccases or a substrate to catalases.

Regarding the LPMOs, only one predicted AA10 enzyme (Pfam *LPMO_10*) was found in the xylan and glucose conditions, suggesting AA3 and AA7 as electron donors. The results also showed that the AA9 enzymes were found in all conditions but were most abundant in the SCB condition, in which AA8 (CDH) could act as electron donor. Interestingly, one AA9 (ANID_02388) was reported in all three conditions; however, this specific enzyme was not reported in the time course secretome of *A. nidulans* grown on sorghum stover for 14 days [42]. The other four AA9 were only found in the SCB condition in high abundance and to a lower extent in the glucose condition; however, they were not found in the xylan condition. These data suggest that AA9 and AA10 enzymes were secreted according to the substrate composition, as some AA9 enzymes were specific for certain growth conditions, suggesting again a different regulation in the production of these enzymes, as well as for AA3 and AA8. Although our data showed that partners LPMO/CDH-GOOX always occurred together, we could not discard the role of lignin as electron donor for the LPMOs [52, 53]. *Aspergillus* species employ significantly different approaches to degrade plant biomass, despite their similar genomic potential. Benoit et al. [54] showed that the significant differences between the enzyme sets produced on wheat bran and sugar beet pulp largely correlated with their polysaccharide composition. The data suggest the conservation of β-glucosidase, cellobiohydrolase, β-galactosidase, β-xylosidase and α-arabinofuranosidase among eight species of *Aspergillus,* highlighting the importance of this group of enzymes to the degradation mechanism in this genus.

### N-glycosylation occurs preferentially at the N-X-T sequon

The sequons present in proteins are strictly targeted for N-glycosylation, as the majority have an N-glycan attached [11]. Thus, we asked if there were patterns or preferences for N-glycosylation in the *A. nidulans* proteins. Mapping N-glyc sites is not a trivial process and can be performed by glycoprotein- or glycopeptide-level enrichment methods [26, 55, 56]. In this study, the protein-level enrichment method allowed for the identification of 182 N-glyc sites. Nineteen (10.4 %) out of 182 sites were previously predicted as non-glycosylated by the NetNGlyc 1.0 Server (assuming a score <0.5). The NetNGlyc tool was designed to discriminate what sequon will accept the N-glycan in human proteins, validating 86 % of glycosylated and 61 % of non-glycosylated sites in all human proteins tested [57]. Moreover, 23 N-glyc sites predicted by NetNGlyc were not validated by our data set. This result suggests that there is some inaccuracy in the prediction of N-glyc sites in fungal enzymes by NetNGlyc. Despite this small divergence, our data show that the NetNGlyc server was a great tool for predicting glycosylated sequons in *A.nidulans* proteins.

The 151 N-glyc sites with an acceptable “Ascore” [30] clearly showed the predominance of the consensus sequence N-X-T, representing 72.2 % of glycosylated sequons (Table 2), similarly to that described by Petrescu et al. [58]. Furthermore, there were variations of the NXT motif such as NGT (12.6 %), NST (9.3 %) and NTT (7.9 %). In 2010, Rao and Bernd [59] elegantly asked if N-glycoproteins have a preference for specific sequons. To answer this question, these authors analyzed viral, archaeal and eukaryotic sequons with experimentally validated N-glyc sites and detected a preference for some amino acids such as F, G, I, S, T and V in the sequon “X” position, whereas the charged amino acids and proline were found to be represented at a lower level.

### Hydrophobic and polar uncharged amino acids are predominant around N-glycosylated sites

In nature, not all protein sequons (NXT/S) are glycosylated. In *A. nidulans,* approximately 50 % of the amino acids adjacent to N-glycosylated sites were hydrophobic or polar uncharged (Fig. 4). These amino acids are responsible for producing a microenvironment able to receive the carbohydrate from the oligosaccharyl transferase (OST). The N-glycan attached to asparagine affects the local charge, exposing the motif region, and its interaction with the amino acid residues is responsible for decreasing the enzyme’s dynamics and increasing the thermostability, increasing the stability against proteolysis [60, 61].

The presence of hydrophilic N-glycans on the surface of hydrophobic proteins affects primarily the thermostability, dynamics, solubility and secretion [60, 62, 63]. Sagt et al. [65] showed the effect of insertion of N-glyc sites into hydrophobic proteins. The addition of a consensus sequence in the N- or C-terminal region decreased the protein aggregation in the ER and enhanced the secretion by 5- and 1.8-fold, respectively [64]. Hence, the N-glycosylation in hydrophobic regions could be related to an evolutionary process involving protein folding, stability and secretion.

Analyzing glycosylated sequons from proteins in the Protein Data Bank (PDB), Petrescu and collaborators [58] showed the presence of N-glycans attached on different surface geometries. The surface diversity suggests that the N-glycosylation process was carefully selected to occur depending on glycan accessibility. Moreover, a predominance of hydrophobic followed by non-polar amino acid residues was detected [59], corroborating our data, despite the fact that we did not clearly detect a higher frequency of aromatic amino acids before the N-glyc site. The sequon neighborhood has also been studied to improve the stability of target enzymes through biotechnological approaches, for example, by the addition of an aromatic amino acid before the N-glycosylated sequon to increase glycoprotein stability [66, 67].

The amino acids flanking the non-validated N-glyc sites (Additional file 1: Figure S4) were interestingly different from the validated ones.

### The N-glycosylation sites are not completely conserved in homologous sequences

**Fig. 7:**
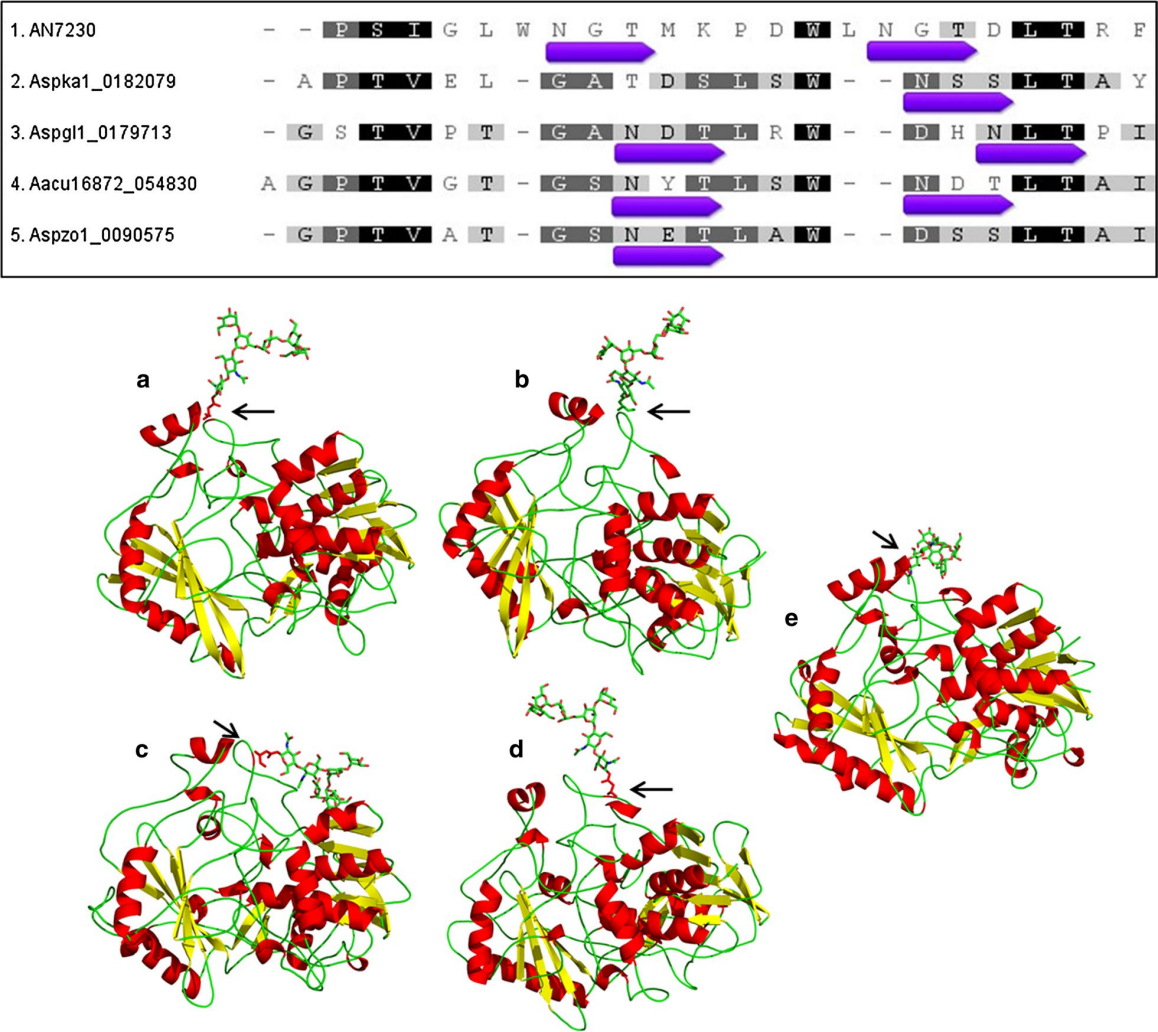
Conservation of N-glycosylation sites in AN7230 and homologous proteins. Four AN7230 homologous sequences were selected to represent the slight differences into cellobiose dehydrogenase enzymes. The sequence alignment shows variation of N-glycosylated sequon position at primary sequence level in some homologous sequences. However, the structures show that the N-glycans are attached in the same loop at 3D-level (*arrows*). Selected proteins are from **a** *A. nidulans* AN7230 **b** *A. kawachii* (Aspka1_0182079) **c** *A. glaucus* (Aspgl1_0179713) **d** *A. aculeatus* (Aacu16872_054830) and **e** *A. zonatus* (Aspzo1_0090575). The 3D protein structures were modeled using the SWISS-MODEL [69] based on *Phanerochaete chrysosporium* cellobiose dehydrogenase (PDB:1KDG)

We asked if the N-glyc sites validated in our data were conserved among *Aspergilli* homologous proteins. This is a central question driving the prediction of N-glycosylation patterns in heterologous proteins to increase heterologous secretion by *Aspergilli* hosts. The analysis of selected N-glyc sites showed variable profiles of conservation at the primary sequence level (Table 3). However, this variation can be a consequence of the alignment, which creates some gaps in the sequences, shifting the sequons among the homologous sequences [68]. For example, the N-glyc sites N299 and N308 were validated by the LC–MS/MS assay in the AA8 cellobiose dehydrogenase (AN7230), as shown in Table 3. AN7230 showed some variations at the N-glycosylated positions at the primary sequence level in a homologous alignment, but at the tridimensional structural level, the glycosylation position was quite similar and occurred in the same protein region (Fig. 7). Several studies have shown that the position of the N-glycan in each protein structure is important due to the influence of the free energy in the region [66, 67].

Tan et al. [61] showed that homologous sequences often have no conserved sequons. The features that guide the N-glycan attachment to the target sequon are extremely complex and, therefore, little is known about this phenomena. Enzymes that lack N-glycosylation sequons in homologous sequences most likely found different evolutionary paths by acquiring mutations that allow for the maintenance of similar characteristics in the microenvironment [70]. However, without a broad study of glycoproteins, it is not possible to affirm if sequences evolved to acquire N-glycosylation sequons or if the attachment of N-glycans was the original event and the sequences are evolving to lack N-glycosylation sites.

### A range of 5–9 mannose residues is predominant in *A. nidulans* N-glycans

We also analyzed the number of mannose residues in N-glycans released from the *A. nidulans*-secreted proteins. The results show that high-mannose N-glycans were predominant, as previously described in *Aspergillus* sp. [33]. It has been reported that *Aspergillus* sp. can carry high-mannose type N-glycans with galactofuranoses [71]. Mannose and galactofuranose are structural isomers, have exactly the same mass and are not distinguishable from oligosaccharide profiling by MALDI/TOF–MS. Thus, in this study, the peaks were labeled as “Hex_5_HexNAc_2_,” but according to the literature, this is likely to be Man_5_GlcNAc_2_ [31, 32]. The genus *Aspergillus* rarely displays hyperglycosylation, and the largest N-glycan was described with 18 mannose residues [32], which is similar to our data. However, we did not detect glucose or galactose residues in the high-mannose glycans as previously reported for *A. niger* [71].

A recent study showed that the difference in N-glycans composition is directly related to the protein structure [72]. Despite the fact that N-glycosylation occurs mainly in β-turns, the sequons could be present in a large variety of structures with low to high accessibility [58, 72]. We found a total of 25, 19 and 40 exclusive proteins in the glucose, SCB and xylan conditions, respectively. Therefore, these specific proteins found in each secretome could explain the divergence of the N-glycans structures (Fig. 6).

Recently, some studies have shown that different substrates can also influence the composition of PTMs. Adav et al. [25] detected changes in the N-glycosylation profile in *Phanerochaete chrysosporium* when grown in glucose, cellulose and lignin. The authors showed that the same protein had differences in the position and number of glycosylation sites depending on the substrate. Moreover, Stals et al. [73] analyzed the N-glycosylation modification in Cel7A from *T. reesei* strains. However, by analyzing the proteins common to all the three growth conditions (99 proteins), we did not detect changes in the profile of N-glycosylation when *A. nidulans* was cultivated in different carbon sources.

The knowledge of the N-glycosylation pattern of secreted proteins can assist in the design of *A. nidulans* as a host for heterologous protein production. However, understanding the N-glycosylation of wild-type enzymes is important, as changes in N-glycan composition can affect the main properties of these enzymes [74]. The N-glycan profile of *T. reesei* has been reported because it is largely used in industry due to its good protein secretion capabilities [75, 76]. The *T. reesei* RUT-C30 strain was reported to have one additional α-1,3-glucose residue in the N-glycan of the main cellobiohydrolase (CBH), suggesting an incorrect maturation process of the N-glycan [76]. The N-glycan composition could interfere at the level of protein secretion. The enzyme secretion process is too complex to affirm that N-glycosylation is the unique feature interfering in the level of protein secretion, although N-glycans have been reported to contribute to the secretion process.

## Conclusions

The knowledge regarding protein glycosylation in a model host such as *A. nidulans* is fundamental to improving the success of heterologous protein secretion. For example, our personal laboratory experience using *A. nidulans* for this purpose has shown that almost all the recombinant genes transformed are overexpressed but only 30 % are effectively translated and secreted (unpublished data).

There are many bottlenecks in protein production by filamentous fungi, such as folding, transport by vesicles, and secretion, but N-glycosylation at the correct sites is a fundamental event to ensure a high level of secretion of target proteins [7, 21, 77, 78]. Our data may assist attempts for the design of glycosylation sites of recombinant genes to be expressed in filamentous fungal hosts. *Aspergillus nidulans* is a model filamentous fungus with an excellent protein secretion system and with a GRAS (generally regarded as safe) status. Although *A. nidulans* is not the main strain used for industrial biomass degradation, it shows a specialized repertoire for biomass degradation compared with other filamentous fungi [51]. Moreover, a large number of genes are specific to *A. nidulans*, when compared with other *Aspergillus* species, and a study of these enzymes could provide advantages [54].

This is the first study to report the N-glycoproteomics of *A. nidulans,* with analysis of proteins, N-glyc sites and N-glycans. Using glucose, xylan and SCB as substrates, we detected 265 proteins strictly related to each substrate, as well as demonstrated different patterns of total proteins, glycoproteins and N-glycan profiles. Glycosylation studies rarely highlight the modifications in CAZymes, which was a focus in this study. *A. nidulans* has a preference for the sequon NXT and specific variations. The results of this study should allow for better manipulation of heterologous proteins using *Aspergillus* spp. as a host.

## Methods

### Media and strain

*Aspergillusnidulans* strain A773 (pyrG89;wA3;pyroA4) was purchased from the Fungal Genetics Stock Center (FGSC). *Aspergillus nidulans* minimal medium (MM) contained salts solution [79] (NaNO_3_ 6 g/L, KCl 0.52 g/L, MgSO_4_·7H_2_O 0.52 g/L, KH_2_PO_4_ 1.52 g/L), trace elements (H_3_BO_3_ 0.011 g/L, MnCl_2_·4H_2_O 0.005 g/L, FeSO_4_·7H_2_O 0.005 g/L, CoCl_2_·6H_2_O 0.0016 g/L, CuSO_4_·5H_2_O 0.0016 g/L, Na_2_MoO_4_·4H_2_O 0.0011 g/L, ZnSO_4_·7H_2_O 0.022 g/L, Na_2_EDTA 0.050 g/L) and was supplemented with pyridoxine (1 mg/L) and uracil/uridine (1.2 g/L each) [80]. 10 g/L of glucose, xylan from beechwood or NaOH-pretreated sugarcane bagasse (SCB) were used as carbon source in different conditions as needed, and pH was adjusted to 6.5 buffered with 200 mM HEPES (4-(2-hydroxyethyl)-1-piperazineethanesulfonic acid) [80].

### Growth conditions

*Aspergillus nidulans* A773 was cultivated in solid minimal media with glucose for 3–4 days; spores were harvest and filtered using Miracloth (Merck Millipore). 10^6^ spores were inoculated into 100 mL MM glucose for 24 h, 37 °C and 180 rpm [80]. The mycelium was collected by filtration, washed using deionized water and transferred to MM containing glucose, SCB (60 % cellulose, 23 % hemicellulose, 8 % lignin and 10 % ash) or xylan for 4 days at the same conditions. Extracellular proteins (secretome) were obtained by filtration of supernatant through one layer of Miracloth. A triplicate was prepared to each secretome. The pretreatment of sugarcane bagasse was carried out as previously described by Rocha et al. [81].

### Enzymatic assays

The polysaccharides xylan from beechwood, xyloglucan from tamarind, mannan, lichenan from *Laminaria digitata*, β-glucan from barley, carboxymethyl cellulose (CMC) and starch were hydrolyzed by *A. nidulans* secretomes produced on sugarcane bagasse and xylan. The enzymatic microassay was carried out using 50 μL of the substrates (0.5 % *w/v*), 50 mM of ammonium acetate buffer at pH 5.5 and 0.5 µg of total protein at 50 °C for 120 min. The reactions were stopped using 100 μL of 3,5-dinitrosalicylic acid (DNS) boiled at 99 °C for 5 min and the reducing sugars were measured at 550 nm. The FPAse activity was performed as recommended by Eveleigh et al. [82] following the modifications proposed by Camassola and Dillon [83]. All the enzymatic assays were performed in triplicate.

### Glycoprotein enrichment and deglycosylation

The secreted proteins (secretome) were concentrated using centrifugal filters with 10 kDa of pore size to obtain 700 μg to 1 mg of total proteins. Glycoproteins in the secretomes were enriched by interaction in Concanavalin A (ConA—GE Healthcare) for 2 h. The glycoproteins were eluted using a buffered-solution of 500 mM methyl α-d-glucopyranoside and the eluted proteins were loaded into a 10 % SDS-PAGE for 30 min at 110 V. The gel bands were excised and treated with 10 units of endoglycosidase-H (Endo H; New England Biolabs) at 37 °C during 24 h for deglycosylation under denaturing conditions.

### Sample preparation for LC–MS/MS analysis

Proteins deglycosylated in-gel were reduced (5 mM dithiothreitol, 30 min, at room temperature), alkylated (14 mM iodoacetamide, 30 min at room temperature in the dark), and digested with 20 mg/mL trypsin (Promega). After peptide extraction, the samples were dried in a vacuum concentrator. 4.5 µL of the peptide mixture was analyzed on an ETD-enabled LTQ Velos Orbitrap mass spectrometer (Thermo Fisher Scientific) coupled with LC–MS/MS by an EASY-nLC system (Proxeon Biosystems) through a Proxeon nanoelectrospray ion source. The peptides were separated by a 2–90 % acetonitrile gradient in 0.1 % formic acid using a PicoFrit Column analytical column (20 cm × ID75 μm, 5 μm particle size, New objective) at a flow rate of 300 nL/min over 60 min. The nanoelectrospray voltage was set to 2.2 kV, and the source temperature was 275 °C. The instrument method for the LTQ Velos Orbitrap was set up in the data-dependent acquisition mode. The full scan MS spectra (*m/z* 300–1600) were acquired in the Orbitrap analyzer after accumulation to a target value of 1e^6^. Resolution in the Orbitrap was set to *r* = 60,000, and the 20 most intense peptide ions with charge states ≥2 were sequentially isolated to a target value of 5000 and fragmented in the linear ion trap by low-energy CID (normalized collision energy of 35 %). The signal threshold for triggering an MS/MS event was set to 1000 counts. Dynamic exclusion was enabled with an exclusion size list of 500, exclusion duration of 60 s, and repeat count of 1. An activation q of 0.25 and an activation time of 10 ms were used.

The raw data files were converted to a peak list format (mgf) using the Mascot Distiller v.2.3.2.0 software (Matrix Science Ltd.). These spectra were searched against the *A. nidulans* genome from AspGD (10.560 entries) using the Mascot v.2.3.01 engine (Matrix Science Ltd.) with oxidation of methionine and *N*-acetylglucosamine (GlcNAc) tagged on asparagine residue (N + 203) as variable modifications, and carbamidomethylation as fixed modification. Additional parameters were one trypsin-missed cleavage, a tolerance of 10 ppm for precursor ions and 1 Da for fragment ions.

### Data analysis

All datasets processed using the workflow feature in the Mascot software were further analyzed in the software ScaffoldQ + (Proteome Software) to validate the MS/MS-based peptide and protein identifications. Peptide identifications were accepted if they could be established at greater than 95 % probability as specified by the Peptide Prophet algorithm [84]. Peptide identifications were also required to exceed specific database search engine thresholds. Mascot identifications required at least both the associated identity scores and ion scores to be *p* < 0.05. Protein identifications were accepted if they could be established at greater than 99 % probability for protein identification. Protein probabilities were assigned using the Protein Prophet algorithm [85]. Proteins that contained similar peptides and could not be differentiated based on the MS/MS analysis alone were grouped to satisfy the principles of parsimony. The scoring parameter (Peptide Probability) in the ScaffoldQ + software obtained a false discovery rate (FDR) of 0.73 %. Using the number of total spectra output from the ScaffoldQ + software, we identified the differentially expressed proteins using spectral counting. Quantitative value was applied to normalize the spectral counts. The Scaffold PTM (Proteome Software) was used to further validate glycosylated sites assignments with confidence, based on their pre-established parameters [30, 86].

### Glycomics

Around 700 μg of each secretome was treated with a mixture of chloroform and methanol by four times, to extract lipids. The extracts were incubated at room temperature with end-over-end agitation. After each lipid extraction procedure, the insoluble protein-containing materials were collected by centrifugation. The final insoluble protein pellets were further washed with cold-acetone/water (4:1, *v/v*) to eliminate polysaccharides from culture media. Pellets were finally washed with cold-acetone and dried under a stream of nitrogen. The dried samples were dissolved in 0.1 M Tris–HCl buffer, pH 8.2 containing 10 mM CaCl_2_ and denatured by heating for 5 min at 100 °C. After cooling, the samples were digested with trypsin (37 °C, overnight). The samples were heated at 100 °C for 5 min to inactivate trypsin and centrifuged at 3000 rpm in a refrigerated centrifuge for 15 min. The supernatants were collected and dried. Samples were then passed through a C18 sep-pak cartridge and washed with 5 % acetic acid to remove contaminants (salts, free sugar, etc.). Peptides and glycopeptides were eluted in series with 20 % iso-propanol in 5 % acetic acid, 40 % iso-propanol in 5 % acetic acid and 100 % iso-propanol and dried in a speed vacuum concentrator. The dried samples were combined and incubated with PNGase F at 37 °C overnight to release N-glycans. After digestion, the samples were passed through a C18 sep-pak cartridge and the released N-glycans were eluted with 5 % acetic acid and dried by lyophilization, and then permethylated based on the method of Anumula and Taylor [87] and profiled by mass spectrometry. MALDI/TOF–MS was performed in the reflector positive ion mode using α-dihyroxybenzoic acid (DHBA, 20 mg/mL solution in 50 % methanol:water) as a matrix. The spectrum was obtained using a TOF/TOF™ 5800 System (AB SCIEX).

## Additional files

10.1186/s13068-016-0580-4 *Aspergillus nidulans* CAZymes inventory. (A) The annotation of *A. nidulans* FGSC A4 CAZymes genes were determined according to CAZy database (http://www.cazy.org/e185.html). The prediction of N-glycosylation sites were carried out using NetNGlyc 1.0 Server that examine the sequence context of Asn-Xaa-Ser/Thr sequons with threshold 0.5. CAZymes predicted with N-glycosylation sites were analyzed for signal peptide using SignalP 4.1 Server. (B) The 190 proteins predicted with N-glycosylation sites and signal peptide were grouped and shown as CAZy families percentage. **Figure S2.** Counts of *A. nidulans* extracellular CAZymes predicted with N-glycosylation. The number of N-glycosylation sites of each CAZyme predicted with signal peptide were analyzed by NetNGlyc 1.0 server. **Figure S3.** Enzymatic repertoire in *A. nidulans* secretomes. Secretomes produced on sugarcane bagasse and xylan were assayed for the hydrolysis of polysaccharides. All the enzymatic assays were carried out at 50 °C for 120 min, using 0.5 µg of total protein. The hydrolysis were performed in triplicate. **Figure S4.** Amino acids distribution around N-glycosylation sites. The relative occurrence of amino acids is plotted versus sequence position −6 to +6 around validated or predicted N-glyc site. Glycosylated: N-glyc sites validated by LC–MS/MS; non-glycosylated: N-glyc sites predicted by NetNGlyc Server but not validated by LC–MS/MS data set. Hydrophobic (Ala, Val, Leu, Ile, Met); Aromatic (Phe, Tyr, Trp); Polar uncharged (Ser, Thr, Asn, Cys, Gln); Acidic (Asp, Glu); Basic (Lys, Arg, His); Unique (Gly, Pro).
10.1186/s13068-016-0580-4 Identified proteins and spectrum counts.
10.1186/s13068-016-0580-4 Identified CAZymes and spectrum counts.
10.1186/s13068-016-0580-4 Identified motifs on N-glycosylated sites.
10.1186/s13068-016-0580-4 N-linked glycans released from glycoproteins secreted by *A. nidulans* detected by MALDI TOF/TOF MS.

## Abbreviations

CAZymes: carbohydrate-active enzymes
PTM: post-translational modifications
SCB: sugarcane bagasse
CBM: carbohydrate-binding module
GHs: glycoside hydrolases
GTs: glycosyltransferases
PLs: polysaccharide lyases
CEs: carbohydrate esterases
AAs: auxiliary activities
ER: endoplasmic reticulum
OST: oligosaccharyltransferases
UPR: unfolded protein response
AspGD: *Aspergillus* Genome Database
N-glyc site: N-glycosylation site
FDR: False Discovery Rate
LPMOs: lytic polysaccharide monooxygenases
CDH: cellobiose dehydrogenases
GOOX: glucooligosaccharide oxidases
PDB: Protein Data Bank
CBH: cellobiohydrolase
GRAS: generally regarded as safe
FGSC: Fungal Genetics Stock Center
MM: minimal medium
CMC: carboxymethyl cellulose
DNS: 3,5-dinitrosalicylic acid
ConA: concanavalin A
Endo H: endoglycosidase-H
GlcNAc: *N*-acetylglucosamine
