# Supplementary material for: Mapping N-linked glycosylation of carbohydrate-active enzymes in the secretome of Aspergillus nidulans grown on lignocellulose
Source: Biotechnol Biofuels. 2016 Aug 8;9:168. doi: 10.1186/s13068-016-0580-4 (PMC4977673; doi:10.1186/s13068-016-0580-4)
Supplement: Supplementary file 1 — 10.1186/s13068-016-0580-4 Aspergillus nidulans CAZymes inventory. (A) The annotation of A. nidulans FGSC A4 CAZymes genes were determined according to CAZy database (http://www.cazy.org/e185.html). The prediction of N-glycosylation sites were carried out using NetNGlyc 1.0 Server that examine the sequence context of Asn-Xaa-Ser/Thr sequons with threshold 0.5. CAZymes predicted with N-glycosylation sites were analyzed for signal peptide using SignalP 4.1 Server. (B) The 190 proteins predicted with N-glycosylation sites and signal peptide were grouped and shown as CAZy families percentage. Figure S2. Counts of A. nidulans extracellular CAZymes predicted with N-glycosylation. The number of N-glycosylation sites of each CAZyme predicted with signal peptide were analyzed by NetNGlyc 1.0 server. Figure S3. Enzymatic repertoire in A. nidulans secretomes. Secretomes produced on sugarcane bagasse and xylan were assayed for the hydrolysis of polysaccharides. All the enzymatic assays were carried out at 50 °C for 120 min, using 0.5 µg of total protein. The hydrolysis were performed in triplicate. Figure S4. Amino acids distribution around N-glycosylation sites. The relative occurrence of amino acids is plotted versus sequence position −6 to +6 around validated or predicted N-glyc site. Glycosylated: N-glyc sites validated by LC–MS/MS; non-glycosylated: N-glyc sites predicted by NetNGlyc Server but not validated by LC–MS/MS data set. Hydrophobic (Ala, Val, Leu, Ile, Met); Aromatic (Phe, Tyr, Trp); Polar uncharged (Ser, Thr, Asn, Cys, Gln); Acidic (Asp, Glu); Basic (Lys, Arg, His); Unique (Gly, Pro). [file 13068_2016_580_MOESM1_ESM.docx]

**A**


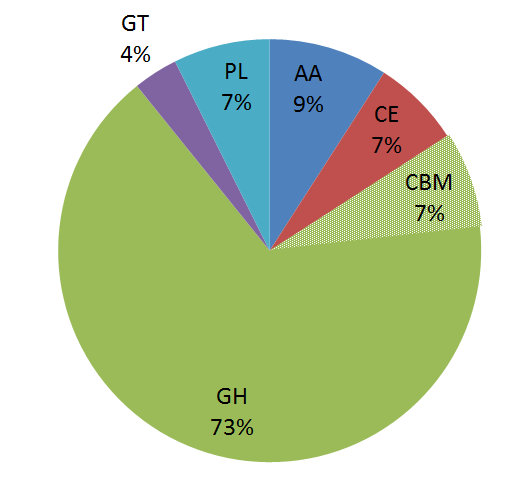


**B**

**Figure S1. *Aspergillus nidulans* CAZymes inventory.**

(A) The annotation of *A. nidulans* FGSC A4 CAZymes genes were determined according to CAZy database (www.cazy.org/e185.html). The prediction of N-glycosylation sites were carried out using NetNGlyc 1.0 Server that examine the sequence context of Asn-Xaa-Ser/Thr sequons with threshold 0.5. CAZymes predicted with N-glycosylation sites were analyzed for signal peptide using SignalP 4.1 Server. (B) The 190 proteins predicted with N-glycosylation sites and signal peptide were grouped and shown as CAZy families percentage.


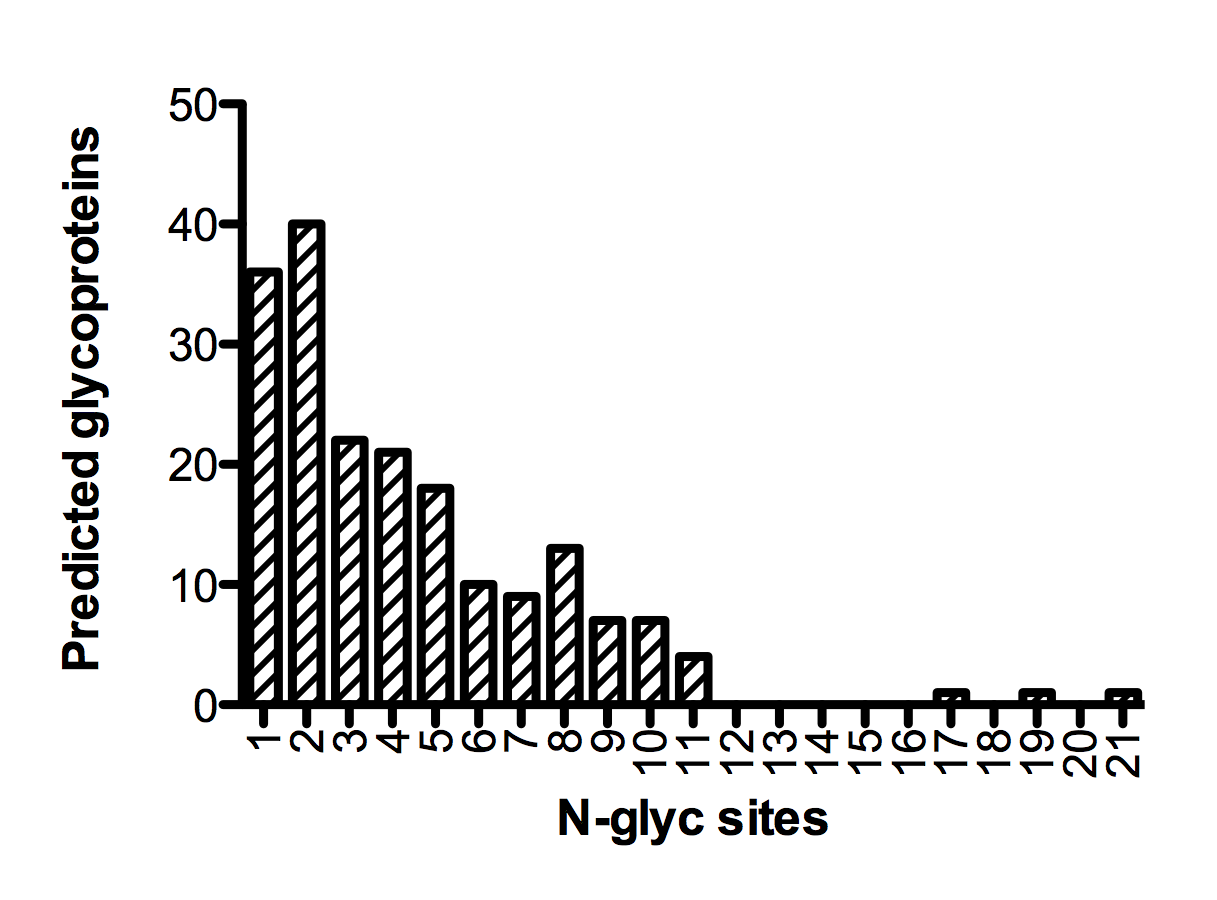


**Figure S2. Counts of *A. nidulans* extracellular CAZymes predicted with N-glycosylation.** The number of N-glycosylation sites of each CAZyme predicted with signal peptide were analyzed by NetNGlyc 1.0 server.

**

**

**Figure S3. Enzymatic repertoire in *A. nidulans* secretomes.**

Secretomes produced on sugarcane bagasse and xylan were assayed for the hydrolysis of polysaccharides. All the enzymatic assays were carried out at 50°C for 120 min, using 0.5 µg of total protein. The hydrolysis were performed in triplicate.

**Figure S4. Amino acids distribution around N-glycosylation sites.** The relative ocurrence of amino acids is plotted versus sequence position -6 to +6 around validated or predicted N-glyc site. Glycosylated: N-glyc sites validated by LC-MS/MS; non-glycosylated: N-glyc sites predicted by NetNGlyc Server but not validadted by LC-MS/MS data set. Hydrophobic (Ala, Val, Leu, Ile, Met); Aromatic (Phe, Tyr, Trp); Polar uncharged (Ser, Thr, Asn, Cys, Gln); Acidic (Asp, Glu); Basic (Lys, Arg, His); Unique (Gly, Pro).
